# Experimental realization of a three-photon asymmetric maximally entangled state and its application to quantum state transfer

**DOI:** 10.1126/sciadv.adj9251

**Published:** 2024-06-21

**Authors:** Linxiang Zhou, Qiao Xu, Tianfeng Feng, Xiaoqi Zhou

**Affiliations:** ^1^ State Key Laboratory of Optoelectronic Materials and Technologies and School of Physics, Sun Yat-sen University, Guangzhou 510000, People’s Republic of China.; ^2^ Hefei National Laboratory, University of Science and Technology of China, Hefei 230088, People’s Republic of China.

## Abstract

Quantum entanglement is crucial for quantum information processing, prominently used in quantum communication, computation, and metrology. Recent studies have shifted toward high-dimensional entangled states, offering greater information capacity and enabling more complex applications. Here, we experimentally prepared a three-photon asymmetric maximally entangled state, comprising two two-dimensional photons and one four-dimensional photon. Using this state, we conducted a proof-of-principle experiment, successfully transferring a four-dimensional quantum state from two photons to another photon with fidelities ranging from 0.78 to 0.86. These results exceed theoretical limits, demonstrating genuine four-dimensional quantum state transfer. The asymmetric entangled state demonstrated here holds promise for future quantum networks as a quantum interface facilitating information transfer across quantum systems with different dimensions.

## Teaser

The asymmetric entangled state enables quantum state transfer across quantum systems of varying dimensions.

## INTRODUCTION

Quantum entanglement (*1*, *2*) is one of the most fundamental and distinctive features of quantum mechanics with many applications in the field of quantum information science, such as quantum state transfer (QST) (*3*–*8*), quantum metrology (*9*–*10*), and quantum communication (*11*–*14*). The most basic and fundamental quantum entangled state is the two-particle, two-dimensional state $\frac{1}{\sqrt{2}}\left(|00\rangle +|11\rangle \right)$, which can be used to show the contradiction between quantum mechanics and local realism (*15*–*17*) and as a physical resource for realizing quantum teleportation of a two-dimensional quantum state. More advanced quantum applications require more complex quantum entangled states. To increase the complexity of quantum entangled states, researchers have worked in two directions: increasing the number of particles and increasing the dimensionality of the particles. Take optical system as an example, by increasing the number of photons, a variety of multi-photon two-dimensional entangled states have been prepared, such as 10-photon (*18*, *19*) and 12-photon entangled states (*20*). In terms of increasing the dimensionality of photons, a variety of two-photon high-dimensional entangled states have also been generated (*21*–*24*), such as the 100 × 100 orbital angular momentum entangled state (*25*). By using such high-dimensional entangled states as the physical resources, quantum teleportation of a high-dimensional quantum state has also been demonstrated (*26*, *27*).

To further increase the complexity of quantum entangled states, attempts have been made to increase the number and dimensionality of particles simultaneously, i.e., to prepare multi-particle high-dimensional quantum entangled states. So far, several works on three-particle high-dimensional entangled states have been reported, including the preparation of the (3, 3, 2) state $\frac{1}{\sqrt{3}}\left(|000\rangle +|111\rangle +|221\rangle \right)$ (*28*), the (3, 3, 3) state $\frac{1}{\sqrt{3}}\left(|000\rangle +|111\rangle +|222\rangle \right)$ (*29*), and the (4, 4, 2) state $\frac{1}{2}\left(|000\rangle +|111\rangle +|220\rangle +|331\rangle \right)$ (*30*), which hold promise for applications in some special quantum communication networks.

Here, we report the preparation of a three-photon asymmetric maximally entangled state, which is the (2, 2, 4) state $\frac{1}{2}\left(|000\rangle +|011\rangle +|102\rangle +|113\rangle \right)$. Such asymmetric maximally entangled state can be used to realize QST between particles of different dimensions, such as transferring an unknown four-dimensional quantum state from two two-dimensional particles to a four-dimensional particle and vice versa. In addition to preparing this asymmetric maximally entangled state, we have also performed a proof-of-principle experiment using it as the physical resource to demonstrate the QST from two qubits to a ququart. Our methods have the potential to be applied to future quantum networks for quantum information transfer between quantum objects of different dimensions.

## RESULTS

### The (2, 2, 4) state and its applications on QST

In future quantum networks, the ability to transfer quantum state between spatially separated nodes will be essential. Typically, this QST relies on maximally entangled states as a crucial resource, with quantum teleportation (*8*) playing a central role in enabling these QSTs. However, the deployment of quantum networks faces added complexity when nodes use quantum state carriers of varying dimensions, such as two-dimensional qubits and higher-dimensional qudits (e.g., four-dimensional ququarts). To overcome this challenge, we propose a new type of quantum state, referred to as the asymmetric maximally entangled state, which is designed to bridge quantum systems of varying dimensions and facilitate the transfer of quantum states across these systems.

Before introducing the asymmetric maximally entangled state, we first review the standard maximally entangled state. For example, the (4, 4) state is a standard maximally entangled state consisting of two four-dimensional particles *a* and *b*

$$|4,4\rangle =\frac{1}{2}\left({|00\rangle }_{a,b}+{|11\rangle }_{a,b}+{|22\rangle }_{a,b}+{|33\rangle }_{a,b}\right)$$ (1)

which can be used as a physical resource for QST of a four-dimensional quantum state (*8*).

The (2, 2, 4) state is a related quantum state

$$\begin{array}{cc} |2,2,4\rangle = & \frac{1}{2}({|000\rangle }_{a1,a2,b}+{|011\rangle }_{a1,a2,b}\\ & +{|102\rangle }_{a1,a2,b}+{|113\rangle }_{a1,a2,b}) \end{array}$$ (2)

where *a*1 and *a*2 are two-dimensional particles, and *b* is a four-dimensional particle. Comparing Eq. 2 with Eq. 1, it can be seen that there is a direct correspondence between the (2, 2, 4) state and the (4, 4) state. For the (2, 2, 4) state, if particles *a*1 and *a*2 are regarded as system A and particle *b* is regarded as system B, both A and B are four-dimensional systems, and their composite system is in a four-dimensional maximally entangled state. Since the number of particles in systems A and B are different, we call the (2, 2, 4) state an asymmetric maximally entangled state.

Similar to the symmetric (4, 4) state, the asymmetric (2, 2, 4) state can also be used as a physical resource for QST. By sending particles *a*1 and *a*2 to Alice and particle *b* to Bob, the (2, 2, 4) state can enable QST of a four-dimensional quantum state from Alice to Bob.

**Fig. 1.**
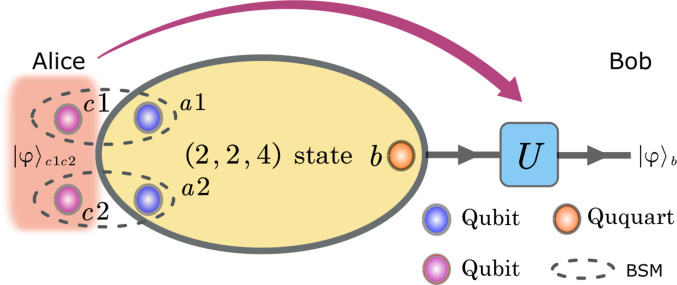
Quantum state transfer protocols based on the (2, 2, 4) state. By sending qubits *a*1 and *a*2 to Alice and ququart *b* to Bob, a quantum state transfer (QST) can be realized between Alice and Bob. If Alice has two qubits *c*1 and *c*2 on which a four-dimensional quantum state ∣φ〉 is encoded, she can make Bell state measurements on qubits *a*1 and *c*1 and on qubits *a*2 and *c*2, respectively, and then send the measurement results to Bob. On the basis of the measurement results, Bob then performs the corresponding unitary transformation on *b*, thus realizing the QST of the four-dimensional quantum state ∣φ〉 from *c*1 and *c*2 to *b*.

As shown in Fig. 1, suppose Alice has two qubits *c*1 and *c*2 in an unknown quantum state

$$\alpha {|00\rangle }_{c1,c2}+\beta {|01\rangle }_{c1,c2}+\gamma {|10\rangle }_{c1,c2}+\delta {|11\rangle }_{c1,c2}$$ (3)

which can be either a separable or entangled state. The joint quantum state of *c*1, *c*2, *a*1, *a*2, and *b* can thus be written as

$$\begin{array}{c} \left(\alpha {|00\rangle }_{c1,c2}+\beta {|01\rangle }_{c1,c2}+\gamma {|10\rangle }_{c1,c2}+\delta {|11\rangle }_{c1,c2}\right)\\ ⊗\frac{1}{2}\left({|000\rangle }_{a1,a2,b}+{|011\rangle }_{a1,a2,b}+{|102\rangle }_{a1,a2,b}+{|113\rangle }_{a1,a2,b}\right)\\ ={|\Phi^{+}\rangle }_{c1,a1}{|\Phi^{+}\rangle }_{c2,a2}⊗\left(\alpha {|0\rangle }_{b}+\beta {|1\rangle }_{b}+\gamma {|2\rangle }_{b}+\delta {|3\rangle }_{b}\right)\\ +{|\Phi^{+}\rangle }_{c1,a1}{|\Phi^{-}\rangle }_{c2,a2}⊗\left(\alpha {|0\rangle }_{b}-\beta {|1\rangle }_{b}+\gamma {-|2\rangle }_{b}-\delta {|3\rangle }_{b}\right)\\ \cdots \cdots +{|\Psi^{-}\rangle }_{c1,a1}{|\Psi^{+}\rangle }_{c2,a2}⊗\left(-\delta {|0\rangle }_{b}-\gamma {|1\rangle }_{b}+\beta {|2\rangle }_{b}+\alpha {|3\rangle }_{b}\right)\\ +{|\Psi^{-}\rangle }_{c1,a1}{|\Psi^{-}\rangle }_{c2,a2}⊗\left(\delta {|0\rangle }_{b}-\gamma {|1\rangle }_{b}-\beta {|2\rangle }_{b}+\alpha {|3\rangle }_{b}\right) \end{array}$$ (4)

where $|\Phi^{\pm }\rangle =\frac{1}{\sqrt{2}}\left(|0\rangle |0\rangle \pm |1\rangle |1\rangle \right)$ and $|\Psi^{\pm }\rangle =\frac{1}{\sqrt{2}}(|0\rangle |1\rangle \pm |1\rangle |0\rangle )$. Alice performs Bell state measurements on two sets of qubits: *c*1 and *a*1, as well as *c*2 and *a*2, respectively. She then sends the measurement outcomes to Bob via a classical channel. Bob then performs the corresponding local unitary operation on particle *b* based on the measurement results, thus obtaining

$$\alpha {|0\rangle }_{b}+\beta {|1\rangle }_{b}+\gamma {|2\rangle }_{b}+\delta {|3\rangle }_{b}$$ (5)

Comparing Eq. 5 with Eq. 3, it can be seen that the two quantum states have exactly the same form except for the difference in the state basis, which indicates that the unknown quantum state with two qubits of quantum information has been successfully transferred from *c*1 and *c*2 to *b*.

Similarly, Bob can also use this (2, 2, 4) state as a resource to transfer a four-dimensional quantum state from a ququart to two qubits (see Supplementary Materials section S1).

### Optical qubit-ququart controlled gate

**Fig. 2.**
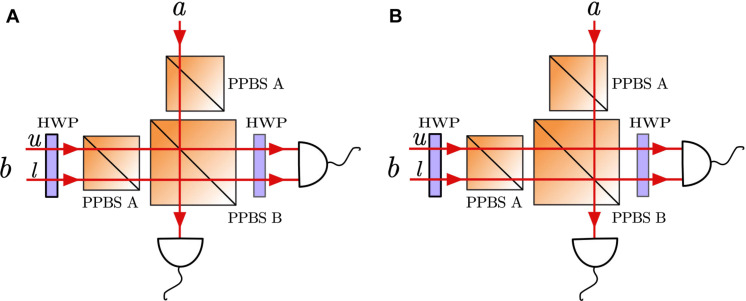
Qubit-ququart controlled gate. Photon *a* is encoded in a two-dimensional quantum state using its polarization. Photon *b* is encoded in a four-dimensional quantum state, using both polarization and spatial degrees of freedom. These photons pass through an optical device comprising three partially polarizing beam splitters (PPBSs) and two half-wave plates (HWPs) set at 22.5^∘^. Through post-selection measurement, this optical device can implement a qubit-ququart controlled gate with a success probability of 1/9. (**A**) Photon *a* and photon *b*’s upper path intersect at the PPBS B, where the optical device subsequently implements a qubit-ququart controlled gate. The function of this gate is as follows: If photon *a* is horizontally polarized, the quantum state of photon *b* remains unchanged; if photon *a* is vertically polarized, the polarization of photon *b*’s upper path is reversed, while its lower path’s polarization remains unaffected. (**B**) Photon *a* and photon *b*’s lower path intersect at the PPBS B, where the optical device subsequently implements a qubit-ququart controlled gate. The function of this gate is as follows: If photon *a* is horizontally polarized, the quantum state of photon *b* remains unchanged; if photon *a* is vertically polarized, the polarization of photon *b*’s lower path is reversed, while its upper path’s polarization remains unaffected.

Let us first explain how to implement a controlled gate on a qubit and a ququart using linear optics. It is recognized that an optical device consisting of three partial polarization beamsplitters (PPBSs) and two half-wave plates (HWPs), as depicted in Fig. 2A, can execute a polarization-encoded two-qubit controlled-NOT (CNOT) operation (*31*–*33*). Here, we will further establish that this optical device is capable of realizing a controlled operation between a qubit and a ququart. Figure 2A illustrates photon *a* as a qubit in the initial state *a*_0_ ∣*H*〉 + *a*_1_ ∣*V*〉, with its quantum information encoded in the polarization degree of freedom (DoF), where *H* (*V*) denotes the horizontal (vertical) polarization. Photon *b*, a ququart, starts in the state *b*_0_∣*Hu*〉 + *b*_1_∣*Hl*〉 + *b*_2_∣*Vu*〉 + *b*_3_∣*Vl*〉, encoding its quantum information across both polarization and path DoFs, where *Hu* (*Hl*) denotes horizontal polarization in the upper (lower) path, and *Vu* (*Vl*) denotes vertical polarization in the upper (lower) path. Photon *a* and photon *b* then pass through the optical device consisting of three PPBSs and two HWPs set at 22.5°. These three PPBSs include two type-A PPBSs and one type-B PPBS, where type-A PPBS perfectly transmits horizontally polarized light while reflecting ^2^/_3_ and transmitting ^1^/_3_ of vertically polarized light, and type-B PPBS perfectly reflects vertically polarized light while transmitting ^2^/_3_ and reflecting ^1^/_3_ of horizontally polarized light. Intersection of photon *a* with the upper path of photon *b* at the PPBS B, along with the detection of exactly one photon at each of the two output ports, enables the optical device to perform a qubit-ququart controlled operation between qubit *a* and ququart *b*. Specifically, for photon *b*’s upper path component *b*_0_∣*Hu*〉 + *b*_2_∣*Vu*〉, the optical device functions to implement a polarization-encoded CNOT operation on qubit *a* and this component; for photon *b*’s lower path component *b*_1_∣*Hl*〉 + *b*_3_∣*Vl*〉, the optical device functions to implement an identity operation on qubit *a* and this component.

Considering the previous analysis, the initial state of qubit *a* and ququart *b*

$$\begin{array}{c} \left(a_{0}|H\rangle +a_{1}|V\rangle \right)⊗\left(b_{0}|\mathrm{Hu}\rangle +b_{1}|\mathrm{Hl}\rangle +b_{2}|\mathrm{Vu}\rangle +b_{3}|\mathrm{Vl}\rangle \right)\\ =(a_{0}|H\rangle +a_{1}|V\rangle )⊗\left(b_{0}|\mathrm{Hu}\rangle +b_{2}|\mathrm{Vu}\rangle \right)\\ +(a_{0}|H\rangle +a_{1}|V\rangle )⊗\left(b_{1}|\mathrm{Hl}\rangle +b_{3}|\mathrm{Vl}\rangle \right) \end{array}$$ (6)

will change to

$$\begin{array}{c} a_{0}|H\rangle \left(b_{0}|\mathrm{Hu}\rangle +b_{2}|\mathrm{Vu}\rangle \right)+a_{1}|V\rangle \left(b_{0}|\mathrm{Vu}\rangle +b_{2}|\mathrm{Hu}\rangle \right)\\ +a_{0}|H\rangle \left(b_{1}|\mathrm{Hl}\rangle +b_{3}|\mathrm{Vl}\rangle \right)+a_{1}|V\rangle \left(b_{1}|\mathrm{Hl}\rangle +b_{3}|\mathrm{Vl}\rangle \right)\\ =a_{0}|H\rangle \left(b_{0}|\mathrm{Hu}\rangle +b_{1}|\mathrm{Hl}\rangle +b_{2}|\mathrm{Vu}\rangle +b_{3}|\mathrm{Vl}\rangle \right)\\ +a_{1}|V\rangle \left(b_{2}|\mathrm{Hu}\rangle +b_{1}|\mathrm{Hl}\rangle +b_{0}|\mathrm{Vu}\rangle +b_{3}|\mathrm{Vl}\rangle \right) \end{array}$$ (7)

after passing through the optical device. Comparing Eq. 6 with Eq. 7, it can be seen that, when qubit *a* is *H*, the state of ququart *b* remains unchanged; when qubit *a* is *V*, the *Hu* component of ququart *b* changes to *Vu*, and the *Vu* component changes to *Hu*, while the *Hl* and *Vl* components remain unchanged, thus realizing a qubit-ququart controlled operation between *a* and *b*. Similarly, the optical device in Fig. 2B can also achieve a qubit-ququart controlled operation between qubit *a* and ququart *b*—when qubit *a* is *H*, the state of ququart *b* remains unchanged; when qubit *a* is *V*, the *Hl* component of ququart *b* changes to *Vl*, and the *Vl* component changes to *Hl*, while the *Hu* and *Vu* components remain unchanged.

### Experimental results

**Fig. 3.**
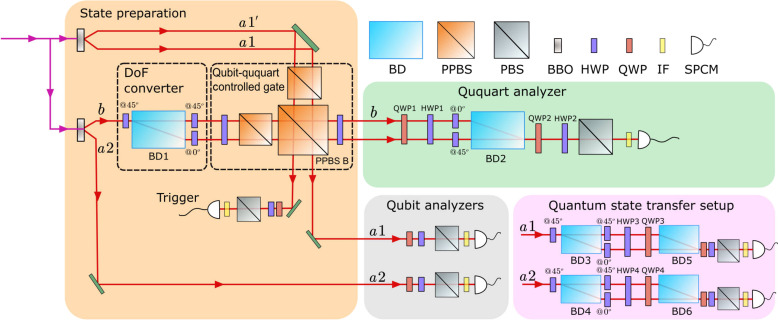
Experimental setup for the preparation of the (2, 2, 4) state. A UV-pulsed laser is focused on two β-barium borate (BBO) crystals to produce two photon pairs *a*1′-*a*1 and *b*-*a*2. After passing through the degree-of-freedom (DoF) converter, photon *b* is split into the upper and lower paths. Photon *b* is first intersected by photon *a*1′ on the PPBS B, and then again intersected by photon *a*1 on the same PPBS B. Each of these intersections activates a qubit-ququart controlled gate, first targeting photons *a*1′ and *b*, and subsequently photons *a*1 and *b*. Photon *a*1′ is then triggered, and the remaining three-photon state consisting of photons *a*1, *a*2, and *b* becomes the desired (2, 2, 4) state. Here, the experimentally obtained fourfold coincidence count rate is 0.183 Hz. To analyze this (2, 2, 4) state, photon *b* is fed to the ququart analyzer (green region) and photons *a*1 and *a*2 are fed to the qubit analyzers (gray region). In the case of performing QST experiment based on the (2, 2, 4) state, photons *a*1 and *a*2 are directly fed into the QST setup (pink region) instead of entering the qubit analyzers. BD, beam displacer; PBS, polarization beamsplitter; QWP, quarter-wave plate; IF, interference filter; SPCM, single-photon counting modules.

After detailing the principles of the qubit-ququart controlled operation, we proceed to describe how we prepare the (2, 2, 4) state experimentally using linear optics. As shown in Fig. 3, a femtosecond-pulsed ultraviolet (UV) laser passes through type-II β-barium borate (BBO) crystals to produce two photon pairs (see Supplementary Materials section S2), photons *a*1′ and *a*1, and photons *b* and *a*2, resulting in the following four-photon state (*20*)

$$\frac{1}{2}\left({|H\rangle }_{a1\prime }{|H\rangle }_{a1}+{|V\rangle }_{a1\prime }{|V\rangle }_{a1}\right)⊗\left({|H\rangle }_{b}{|H\rangle }_{a2}+{|V\rangle }_{b}{|V\rangle }_{a2}\right)$$ (8)

Photon *b* then passes through a device called DoF converter, which consists of a beam displacer (BD1) and the three HWPs before and after it, thus turning *H* into *Hu* and *V* into *Hl*. The four-photon state now becomes

$$\begin{array}{c} \frac{1}{2}\left({|H\rangle }_{a1\prime }{|H\rangle }_{a1}+{|V\rangle }_{a1\prime }{|V\rangle }_{a1}\right)⊗\left({|\mathrm{Hu}\rangle }_{b}{|H\rangle }_{a2}+{|\mathrm{Hl}\rangle }_{b}{|V\rangle }_{a2}\right)\\ =\frac{1}{2}({|H\rangle }_{a1\prime }{|H\rangle }_{a1}{|\mathrm{Hu}\rangle }_{b}{|H\rangle }_{a2}+{|H\rangle }_{a1\prime }{|H\rangle }_{a1}{|\mathrm{Hl}\rangle }_{b}{|V\rangle }_{a2}\\ +{|V\rangle }_{a1\prime }{|V\rangle }_{a1}{|\mathrm{Hu}\rangle }_{b}{|H\rangle }_{a2}+{|V\rangle }_{a1\prime }{|V\rangle }_{a1}{|\mathrm{Hl}\rangle }_{b}{|V\rangle }_{a2}) \end{array}$$ (9)

The upper (lower) path of photon *b* is then superposed with photon *a*1′ (*a*1) on the PPBS B. As explained previously, this PPBS, together with the adjacent two PPBSs and two HWPs, enables a qubit-ququart controlled operation between photons *a*1′ (*a*1) and *b*. Consequently, ququart *b* interacts sequentially with qubit *a*1′ and qubit *a*1. When qubit *a*1′ is *V*, it triggers a polarization reversal in the upper path of ququart *b*. Likewise, when qubit *a*1 is *V*, a polarization reversal occurs in the lower path of ququart *b*. Since the quantum states of qubits *a*1′ and *a*1 only contain the components ∣*HH*〉 and ∣*VV*〉, when qubits *a*1′ and *a*1 are in the ∣*HH*〉 state, the quantum state of ququart *b* remains unchanged, and when qubits *a*1′ and *a*1 are in the ∣*VV*〉 state, the polarization in both the upper and lower paths of ququart *b* undergoes a reversal. Consequently, after passing through these two qubit-ququart controlled gates, the four-photon state becomes

$$\begin{array}{c} \frac{1}{2}({|H\rangle }_{a1\prime }{|H\rangle }_{a1}{|\mathrm{Hu}\rangle }_{b}{|H\rangle }_{a2}+{|H\rangle }_{a1\prime }{|H\rangle }_{a1}{|\mathrm{Hl}\rangle }_{b}{|V\rangle }_{a2}\\ +{|V\rangle }_{a1\prime }{|V\rangle }_{a1}{|\mathrm{Vu}\rangle }_{b}{|H\rangle }_{a2}+{|V\rangle }_{a1\prime }{|V\rangle }_{a1}{|\mathrm{Vl}\rangle }_{b}{|V\rangle }_{a2}) \end{array}$$ (10)

Photon *a*1′ is then projected to $\frac{1}{\sqrt{2}}\left(|H\rangle +|V\rangle \right)$ and the remaing three-photon state thus becomes

$$\begin{array}{c} \frac{1}{2}({|H\rangle }_{a1}{|H\rangle }_{a2}{|\mathrm{Hu}\rangle }_{b}+{|H\rangle }_{a1}{|V\rangle }_{a2}{|\mathrm{Hl}\rangle }_{b}\\ +{|V\rangle }_{a1}{|H\rangle }_{a2}{|\mathrm{Vu}\rangle }_{b}+{|V\rangle }_{a1}{|V\rangle }_{a2}{|\mathrm{Vl}\rangle }_{b}) \end{array}$$ (11)

By defining ∣*H*〉 ≡ ∣0〉 and ∣*V*〉 ≡ ∣1〉 for photons *a*1 and *a*2, and ∣*Hu*〉 ≡ ∣0〉, ∣*Hl*〉 ≡ ∣1〉, ∣*Vu*〉 ≡ ∣2〉, and ∣*Vl*〉 ≡ ∣3〉 for photon *b*, the prepared three-photon state can be rewritten as

$$\frac{1}{2}\left({|000\rangle }_{a1,a2,b}+{|011\rangle }_{a1,a2,b}+{|102\rangle }_{a1,a2,b}+{|113\rangle }_{a1,a2,b}\right)$$ (12)

which is exactly our desired (2, 2, 4) state.

Note that in our experiment, we used qubit-ququart controlled gates based on optical post-selection measurements to apply operations to photon *a*1′ and photon *b*, as well as to photon *a*1 and photon *b* (the overall success rate for the two quantum gates is 1/27), thereby successfully generating a high-dimensional four-photon entangled state. Photon *a*1′ is then projected onto $\frac{1}{\sqrt{2}}\left(|H\rangle +|V\rangle \right)$ (with a success probability of 1/2), resulting in the desired (2, 2, 4) state being produced at an overall success rate of 1/54. If photon *a*1′ were instead measured in the $\frac{1}{\sqrt{2}}\left(|H\rangle \pm |V\rangle \right)$ basis and subjected to a *Z* operation for the $\frac{1}{\sqrt{2}}\left(|H\rangle -|V\rangle \right)$ outcome, the (2, 2, 4) state’s preparation success rate could improve to 1/27. Notably, our experimental implementation of the qubit-ququart gate did not involve ancillary photons. Introducing ancillary photons could allow the use of setup similar to those described in (*34*), enabling the successful implementation of the qubit-ququart gate with a heralding probability of 1/4, and the preparation of the (2, 2, 4) state with a heralding probability of 1/16.

**Fig. 4.**
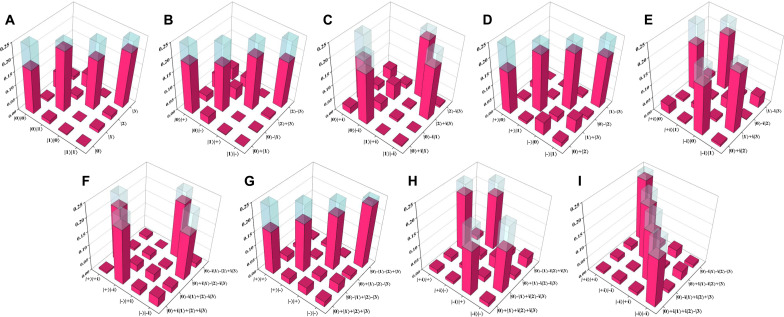
The measurement results of the prepared three-photon state at nine different bases. The fidelity of the prepared state with respect to the ideal (2, 2, 4) state can be calculated from these measurement results (**A **to **I**) as 0.72 ± 0.02. The measurement duration for each set of basis was 2900 s.

To evaluate the fidelity of the prepared (2, 2, 4) state, we let photon *b* enter a device (green region) for measuring ququart states (see Supplementary Materials section S3), and photons *a*1 and *a*2 enter a device (gray region) for measuring qubit states. We then performed measurements on the prepared three-photon state under nine different measurement bases (see Supplementary Materials section S4), and the results are shown in Fig. 4. On the basis of these measurement results, the fidelity of the prepared three-photon state with respect to the ideal (2, 2, 4) state can be calculated to be 0.72 ± 0.02.

After preparing the (2, 2, 4) state, we use it as a resource to perform a proof-of-principle QST experiment. As shown in Fig. 3, instead of sending photons *a*1 and *a*2 for qubit measurements (gray region), we send them into the QST setup (pink region). Photon *a*1 (*a*2) passes through a DoF converter consisting of a beam displacer BD3 (BD4) and the three HWPs before and after it, which expand the dimensionality of photon *a*1 (*a*2) from two to four dimensions, thus turning the three-photon state into

$$\begin{array}{c} \frac{1}{2}({|\mathrm{Hu}\rangle }_{a1}{|\mathrm{Hu}\rangle }_{a2}{|\mathrm{Hu}\rangle }_{b}+{|\mathrm{Hu}\rangle }_{a1}{|\mathrm{Hl}\rangle }_{a2}{|\mathrm{Hl}\rangle }_{b}\\ +{|\mathrm{Hl}\rangle }_{a1}{|\mathrm{Hu}\rangle }_{a2}{|\mathrm{Vu}\rangle }_{b}+{|\mathrm{Hl}\rangle }_{a1}{|\mathrm{Hl}\rangle }_{a2}{|\mathrm{Vl}\rangle }_{b}) \end{array}$$ (13)

If the polarization and path DoFs of photons *a*1 and *a*2 are each written separately as a qubit, this three-photon state can be rewritten as

$$\begin{array}{c} {|H\rangle }_{a1}{|H\rangle }_{a2}⊗\frac{1}{2}({|u\rangle }_{a1}{|u\rangle }_{a2}{|\mathrm{Hu}\rangle }_{b}+{|u\rangle }_{a1}{|l\rangle }_{a2}{|\mathrm{Hl}\rangle }_{b}\\ +{|l\rangle }_{a1}{|u\rangle }_{a2}{|\mathrm{Vu}\rangle }_{b}+{|l\rangle }_{a1}{|l\rangle }_{a2}{|\mathrm{Vl}\rangle }_{b}) \end{array}$$ (14)

where *u* and *l* denote photons in the upper and lower paths, respectively. Photon *a*1 (*a*2) then passes through HWP3 and quarter-wave plate QWP3 (HWP4 and QWP4), and the two polarization qubits of photons *a*1 and *a*2 are then prepared to

$$\alpha {|H\rangle }_{a1}{|H\rangle }_{a2}+\beta {|H\rangle }_{a1}{|V\rangle }_{a2}+\gamma {|V\rangle }_{a1}{|H\rangle }_{a2}+\delta {|V\rangle }_{a1}{|V\rangle }_{a2}$$ (15)

which is the two-qubit state that is set to be transferred. We note that in our experiment, we encode the four-dimensional quantum state to be transferred on the polarization qubits of photon *a*1 and photon *a*2, instead of using separate photons, due to the limited photon counting rate. The path qubits of photon *a*1 and photon *a*2 are then used as the two qubits in the (2, 2, 4) state, serving as a resource for QST (*3*–*6*). Photon *b* corresponds to the ququart in the (2, 2, 4) state.

The joint quantum state of photons *a*1, *a*2, and *b* can now be written as

$$\begin{array}{c} (\alpha {|H\rangle }_{a1}{|H\rangle }_{a2}+\beta {|H\rangle }_{a1}{|V\rangle }_{a2}\\ +\gamma {|V\rangle }_{a1}{|H\rangle }_{a2}+\delta {|V\rangle }_{a1}{|V\rangle }_{a2})\\ ⊗\frac{1}{2}({|u\rangle }_{a1}{|u\rangle }_{a2}{|\mathrm{Hu}\rangle }_{b}+{|u\rangle }_{a1}{|l\rangle }_{a2}{|\mathrm{Hl}\rangle }_{b}\\ +{l\rangle }_{a1}{|u\rangle }_{a2}{|\mathrm{Vu}\rangle }_{b}+{|l\rangle }_{a1}{|l\rangle }_{a2}{|\mathrm{Vl}\rangle }_{b}) \end{array}$$ (16)

It can be seen that there is a one-to-one correspondence between Eq. 16 and Eq. 4. Therefore, according to the previous theoretical derivation, implementing Bell state measurements of the polarization and path qubits of photons *a*1 and *a*2, respectively, and then sending the results to Bob for him to transform photon *b* accordingly can realize the desired QST. Implementing a full deterministic QST requires the use of active feed-forward; however, in this proof-of-principle experiment, we did not apply feed-forward but used post-selection to realize a probabilistic QST.

We let photon *a*1 (*a*2) pass through BD5 (BD6), the subsequent wave plates and the polarization beamsplitter (PBS) to be detected, thus realizing the projection of the polarization and path qubits of photon *a*1 (*a*2) onto the Bell state $\frac{1}{\sqrt{2}}\left({|H\rangle }_{a1}{|u\rangle }_{a1}+{|V\rangle }_{a1}{|l\rangle }_{a1}\right)$ ($\frac{1}{\sqrt{2}}\left({|H\rangle }_{a2}{|u\rangle }_{a2}+{|V\rangle }_{a2}{|l\rangle }_{a2}\right)$). After completing the projection of photons *a*1 and *a*2, the quantum state of photon *b* becomes

$$\alpha {|\mathrm{Hu}\rangle }_{b}+\beta {|\mathrm{Hl}\rangle }_{b}+\gamma {|\mathrm{Vu}\rangle }_{b}+\delta {|\mathrm{Vl}\rangle }_{b}$$ (17)

Comparing Eq. 17 with Eq. 15, it can be seen that the two quantum states have exactly the same form, except for the difference in the state basis, so that the two-qubit state originally encoded in photons *a*1 and *a*2 has been transferred to photon *b*, thus realizing the desired QST. Here, we note that, although the quantum state to be transferred is encoded in the polarization DoF of photons *a*1 and *a*2, the process of the QST does not need prior knowledge about the specific form of this quantum state. The setup determining the quantum state to be transferred involves a series of wave plates (HWP3, QWP3, HWP4, QWP4), whose angular configurations can be adjusted by an external party unassociated with the QST experiment. This arrangement allows the experimental operator to remain unaware of the specific angle settings. Therefore, for the experimental operator, the quantum state to be transferred can be regarded as an unknown four-dimensional quantum state, and in this case, our apparatus remains effective in accurately transferring such unknown quantum state.

In our experiment, we have chosen six different two-qubit states for QST

$$\begin{array}{c} |\varphi_{1}\rangle =\frac{1}{\sqrt{2}}\left({|H\rangle }_{a1}{|V\rangle }_{a2}+{|V\rangle }_{a1}{|V\rangle }_{a2}\right),\\ |\varphi_{2}\rangle =\frac{1}{\sqrt{2}}\left({|H\rangle }_{a1}{|V\rangle }_{a2}-{|V\rangle }_{a1}{|V\rangle }_{a2}\right),\\ |\varphi_{3}\rangle =\frac{1}{2}(({|H\rangle }_{a1}{|H\rangle }_{a2}+{|H\rangle }_{a1}{|V\rangle }_{a2}\\ \;\;+{|V\rangle }_{a1}{|H\rangle }_{a2}+{|V\rangle }_{a1}{|V\rangle }_{a2}),\\ |\varphi_{4}\rangle =\frac{1}{2}({|H\rangle }_{a1}{|H\rangle }_{a2}-{|H\rangle }_{a1}{|V\rangle }_{a2}\\ \;\;+{|V\rangle }_{a1}{|H\rangle }_{a2}-{|V\rangle }_{a1}{|V\rangle }_{a2}),\\ |\varphi_{5}\rangle =\frac{1}{2}({|H\rangle }_{a1}{|H\rangle }_{a2}+i{|H\rangle }_{a1}{|V\rangle }_{a2}\\ \;\;+{|V\rangle }_{a1}{|H\rangle }_{a2}+i{|V\rangle }_{a1}{|V\rangle }_{a2}),\\ |\varphi_{6}\rangle =\frac{1}{2}(({|H\rangle }_{a1}{|H\rangle }_{a2}-i{|H\rangle }_{a1}{|V\rangle }_{a2}\\ \;\;+{|V\rangle }_{a1}{|H\rangle }_{a2}-i{|V\rangle }_{a1}{|V\rangle }_{a2}) \end{array}$$ (18)

**Fig. 5.**
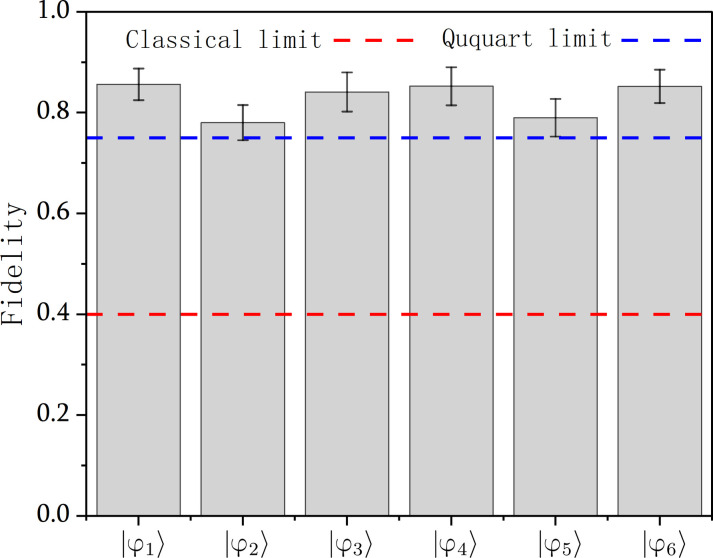
Summary of the fidelities of the QST for the six two-qubit states. The fidelities range from 0.78 to 0.86, which are well above both the optimal single-copy ququart state-estimation limit of 2/5 and maximal qutrit-ququart overlap of 3/4. The error bars (SD) are calculated according to propagated Poissonian counting statistics of the raw detection events. The measurement duration for each of the six two-qubit states was 3900 s.

After completing the QST of ∣φ_1_〉, ∣φ_2_〉, ∣φ_3_〉, ∣φ_4_〉, ∣φ_5_〉, and ∣φ_6_〉, we measured photon *b* at specific state bases to obtain the fidelities of these three QST as 0.86 ± 0.03, 0.78 ± 0.03, 0.84 ± 0.04, 0.85 ± 0.04, 0.79 ± 0.04, and 0.85 ± 0.03, respectively, as shown in Fig. 5 (see Supplementary Materials section S5 for detailed experimental results).

The main sources of error in our experiment include double pair emission, imperfection in preparation of the initial states, and the nonideal interference at the PPBS and BDs. Despite the experimental noise, the fidelities we obtained still far exceed the classical limit of 2/5, which is the best result that can be achieved using classical strategies (*35*, *36*). To demonstrate that the QST of a four-dimensional quantum state is truly accomplished, the transferred quantum state needs to be uninterpretable by a mixture of superposition of three-dimensional or lower-dimensional quantum states, thus requiring that the average fidelity of the transferred quantum states with respect to the ideal states exceeds the ququart limit of 3/4 (*27*). The obtained average fidelity 0.83 ± 0.04 also exceeds this bound, thus decisively proving that we have realized a four-dimensional QST.

## DISCUSSION

To summarize, we have experimentally prepared an asymmetric maximally entangled state consisting of two qubits and one ququart, and used it as a resource to implement a proof-of-principle QST experiment, which realizes the transfer of an unknown four-dimensional quantum state from two photons to another photon. In our work, the asymmetric maximally entangled (2, 2, 4) state used to bridge two-dimensional and four-dimensional quantum systems is realized on a discrete optical system. Our scheme is also applicable to integrated optical platforms and can be extended to higher dimensions. For example, the asymmetric maximally entangled (2, 2, 2, 8) state can be realized on an integrated optical chip (*37*, *38*), which can be used for the QST between two-dimensional and eight-dimensional systems (see Supplementary Materials section S6). In future quantum networks or distributed quantum computing systems, nodes might use quantum information carriers of varying dimensions. Transfers of quantum states or gates between these nodes are frequently required. The asymmetric entangled states demonstrated in this study provide a fundamental quantum resource and interface for enabling the transfer of quantum states or quantum gates (*39*) (see Supplementary Materials section S7) across nodes with quantum information carriers of varying dimensions. The versatility of these states holds potential for widespread deployment in future quantum networks and distributed quantum computing environments.

## MATERIALS AND METHODS

The materials and methods are discussed further in the Supplementary Materials.

## Supplementary Materials

### This PDF file includes:

Sections S1 to S7 Figs. S1 to S6
