# Supplementary material for: Experimental realization of a three-photon asymmetric maximally entangled state and its application to quantum state transfer
Source: Sci Adv. 2024 Jun 21;10(25):eadj9251. doi: 10.1126/sciadv.adj9251 (PMC12697563; doi:10.1126/sciadv.adj9251)
Supplement: Supplementary file 1 — Sections S1 to S7 Figs. S1 to S6 [file sciadv.adj9251_sm.pdf]

Science Advances
AAAS

Supplementary Materials for

Experimental realization of a three-photon asymmetric maximally entangled state and its application to quantum state transfer

Linxiang Zhou *et al.*

Corresponding author: Xiaoqi Zhou, zhouxq8@mail.sysu.edu.cn

Sci. Adv. **10**, eadj9251 (2024)
DOI: 10.1126/sciadv.adj9251

This PDF file includes:

Sections S1 to S7
Figs. S1 to S6

S1. QUANTUM STATE TRANSFER FROM A QUQUART TO TWO QUBITS

As shown in Fig. S1, suppose Bob has a ququart c in an unknown quantum state

$$\alpha|0\rangle_c + \beta|1\rangle_c + \gamma|2\rangle_c + \delta|3\rangle_c, \tag{S1}$$

which contains two qubits of quantum information. The joint quantum state of c, b, $a1$ and $a2$ can thus be written as

$$\begin{aligned}
&(\alpha|0\rangle_c + \beta|1\rangle_c + \gamma|2\rangle_c + \delta|3\rangle_c)\\
\otimes&\frac{1}{2}(|000\rangle_{b,a1,a2} + |101\rangle_{b,a1,a2} + |210\rangle_{b,a1,a2} + |311\rangle_{b,a1,a2})\\
=&|\Phi_{11}\rangle_{c,b}\left(\alpha|00\rangle_{a1,a2} + \beta|01\rangle_{a1,a2} + \gamma|10\rangle_{a1,a2} + \delta|11\rangle_{a1,a2}\right)\\
+&|\Phi_{12}\rangle_{c,b}\left(\alpha|00\rangle_{a1,a2} - \beta|01\rangle_{a1,a2} + \gamma|10\rangle_{a1,a2} - \delta|11\rangle_{a1,a2}\right)\\
&\cdots\cdots\\
+&|\Phi_{44}\rangle_{c,b}\left(\delta|00\rangle_{a1,a2} - \gamma|01\rangle_{a1,a2} - \beta|10\rangle_{a1,a2} + \alpha|11\rangle_{a1,a2}\right),
\end{aligned} \tag{S2}$$

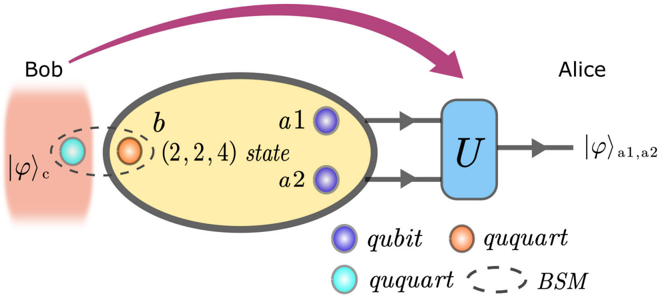

FIG. S1: Quantum state transfer protocols based on the (2, 2, 4) state. By sending qubits $a1$ and $a2$ to Alice and ququart b to Bob, a quantum state transfer can be realized between Alice and Bob. If Bob has a ququart c, he can make a four-dimensional Bell state measurement on ququarts b and c, and then send the measurement result to Alice. Based on the measurement result, Alice then performs the corresponding unitary transformations on $a1$ and $a2$, thus realizing the quantum state transfer from ququart c to the two qubits $a1$ and $a2$.

where $|\Phi_{11}\rangle$ to $|\Phi_{44}\rangle$ are the 16 four-dimensional Bell states (*14*) , which are defined as

$$\begin{aligned}
|\Phi_{11}\rangle &= \frac{1}{2}\left(|00\rangle + |11\rangle + |22\rangle + |33\rangle\right)\\
|\Phi_{12}\rangle &= \frac{1}{2}\left(|00\rangle - |11\rangle + |22\rangle - |33\rangle\right)\\
|\Phi_{13}\rangle &= \frac{1}{2}\left(|00\rangle + |11\rangle - |22\rangle - |33\rangle\right)\\
|\Phi_{14}\rangle &= \frac{1}{2}\left(|00\rangle - |11\rangle - |22\rangle + |33\rangle\right)\\
|\Phi_{21}\rangle &= \frac{1}{2}\left(|01\rangle + |10\rangle + |23\rangle + |32\rangle\right)\\
|\Phi_{22}\rangle &= \frac{1}{2}\left(|01\rangle - |10\rangle + |23\rangle - |32\rangle\right)\\
|\Phi_{23}\rangle &= \frac{1}{2}\left(|01\rangle + |10\rangle - |23\rangle - |32\rangle\right)\\
|\Phi_{24}\rangle &= \frac{1}{2}\left(|01\rangle - |10\rangle - |23\rangle + |32\rangle\right)\\
|\Phi_{31}\rangle &= \frac{1}{2}\left(|02\rangle + |13\rangle + |20\rangle + |31\rangle\right)
\end{aligned} \tag{S3}$$

$$|\Phi_{32}\rangle = \frac{1}{2}\left(|02\rangle - |13\rangle + |20\rangle - |31\rangle\right)$$
$$|\Phi_{33}\rangle = \frac{1}{2}\left(|02\rangle + |13\rangle - |20\rangle - |31\rangle\right)$$
$$|\Phi_{34}\rangle = \frac{1}{2}\left(|02\rangle - |13\rangle - |20\rangle + |31\rangle\right)$$
$$|\Phi_{41}\rangle = \frac{1}{2}\left(|03\rangle + |12\rangle + |21\rangle + |30\rangle\right)$$
$$|\Phi_{42}\rangle = \frac{1}{2}\left(|03\rangle - |12\rangle + |21\rangle - |30\rangle\right)$$
$$|\Phi_{43}\rangle = \frac{1}{2}\left(|03\rangle + |12\rangle - |21\rangle - |30\rangle\right)$$
$$|\Phi_{44}\rangle = \frac{1}{2}\left(|03\rangle - |12\rangle - |21\rangle + |30\rangle\right).$$

Bob performs a four-dimensional Bell state measurement on the two ququarts c and b in his hands, and sends the measurement result to Alice via classical channel. Alice then performs the corresponding local unitary operations on particles $a1$ and $a2$ based on the measurement result and obtains

$$\alpha|00\rangle_{a1,a2} + \beta|01\rangle_{a1,a2} + \gamma|10\rangle_{a1,a2} + \delta|11\rangle_{a1,a2}, \tag{S4}$$

thus realizing the quantum state transfer of an unknown quantum state from ququart c to the two qubits $a1$ and $a2$.

S2. EXPERIMENTAL PREPARATION OF TWO-PHOTON PAIRS

As shown in Fig. 2 of the main text, a 390nm femtosecond-pulsed UV laser passes through type-II beta-barium borate (BBO) crystals to produce two photon pairs , photons $a1'$ and $a1$, and photons b and $a2$, resulting in the following four-photon state

$$\frac{1}{2}\left(|H\rangle_{a1'}|H\rangle_{a1} + |V\rangle_{a1'}|V\rangle_{a1}\right) \otimes \left(|H\rangle_{b}|H\rangle_{a2} + |V\rangle_{b}|V\rangle_{a2}\right), \tag{S5}$$

where H (V) denotes the horizontal (vertical) polarization.

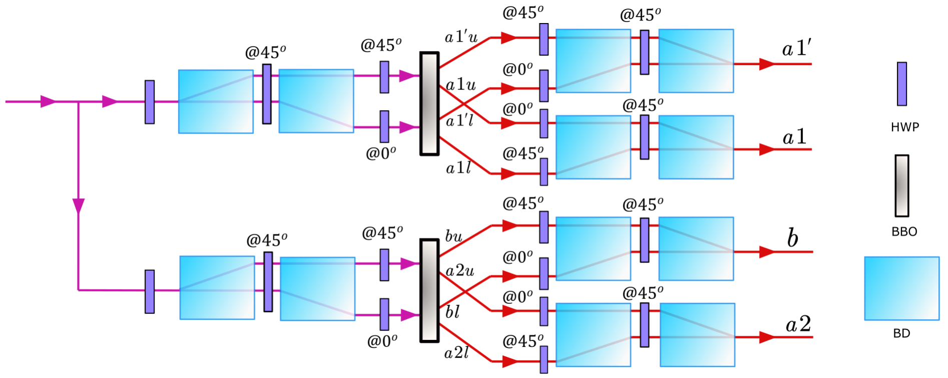

FIG. S2: Experimental setup for generating two photon pairs.

For the sake of simplicity, in Fig. 2 of the main text, we only show a simplified version of the spontaneous parametric down-conversion (SPDC) sources. Figure S2 shows the detailed experimental setup for generating two photon pairs. An ultraviolet pulse laser centered at 390nm is split into two parts, which are used to generate two SPDC photon pairs (*20*). The upper part of the laser goes through a half waveplate (HWP) to prepare its polarization at $\frac{1}{\sqrt{2}}(|H\rangle + |V\rangle)$. It then passes through an arrangement of two beam displacers (BDs) and HWPs to separate the laser into two beams by 4 mm apart . The two beams then focus on a BBO crystal to generate two photon pairs in the states $|H_{a1'u}\rangle|V_{a1u}\rangle$ and $|H_{a1'l}\rangle|V_{a1l}\rangle$ via beamlike type-II SPDC, where the subscripts denote the spatial modes. $|H_{a1'u}\rangle|V_{a1u}\rangle$ and $|H_{a1'l}\rangle|V_{a1l}\rangle$ are then rotated using HWPs to $|V_{a1'u}\rangle|V_{a1u}\rangle$ and $|H_{a1'l}\rangle|H_{a1l}\rangle$, respectively.

Photon pairs of $|V_{a1'u}\rangle\,|V_{a1u}\rangle$ and $|H_{a1'l}\rangle\,|H_{a1l}\rangle$ are then combined into same spatial modes using four BDs and the two HWPs between them. After tilting the BDs to finely tune the relative phase between the two components, the two photons $a1'$ and $a1$ are prepared into $\frac{1}{\sqrt{2}}(|H\rangle_{a1'}|H\rangle_{a1}+|V\rangle_{a1'}|V\rangle_{a1})$. Similarly, the lower part of the laser produces another pair of photons b and $a2$, which are prepared to $\frac{1}{\sqrt{2}}(|H\rangle_b|H\rangle_{a2}+|V\rangle_b|V\rangle_{a2})$.

S3. STATE ANALYSIS OF A PHOTONIC QUQUART STATE

A photon with both polarization and spatial DOFs can encode a ququart state. To fully characterize such state, one needs to perform projective measurement onto various different ququart states. To fulfill this task, we build a ququart state analyzer as shown in Fig. S3, which can project the input ququart to any state in the form of $(a|H\rangle+b|V\rangle)\otimes(c|u\rangle+d|l\rangle)$. This setup works as follows: Suppose the input ququart state is $(a|H\rangle+b|V\rangle)\otimes(c|u\rangle+d|l\rangle)$. After passing through QWP1 and HWP1, which are used to convert $a|H\rangle+b|V\rangle$ to $|H\rangle$, the ququart state becomes $|H\rangle\otimes(c|u\rangle+d|l\rangle)$. The subsequent two HWPs (one at 45° and the other at 0°) and the BD are used to convert $|H\rangle\otimes(c|u\rangle+d|l\rangle)$ to $c|H\rangle+d|V\rangle$, which is now a polarization qubit state. QWP2 and HWP2 are then used to convert $c|H\rangle+d|V\rangle$ to $|H\rangle$, which can pass through the PBS and get detected by the single-photon detector. As a result, for any input ququart state, only its $(a|H\rangle+b|V\rangle)\otimes(c|u\rangle+d|l\rangle)$ component can pass through the setup described above, which effectively realize the desired projective measurement. By changing the parameters a, b, c and d, this state analyzer can be used to perform various projective measurements on the input ququart state.

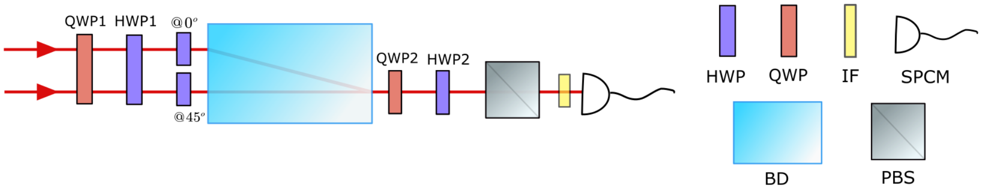

FIG. S3: State analyzer for a single-photon ququart state with both polarization and spatial degrees of freedom.

S4. FIDELITY MEASUREMENT OF THE PREPARED THREE-PHOTON STATE WITH RESPECT TO THE IDEAL (2, 2, 4) STATE

The experimentally prepared three-photon state consisting of photons $a1$, $a2$ and b is

$$\begin{aligned}\frac{1}{2}(&|H\rangle_{a1}|H\rangle_{a2}|Hu\rangle_b+|H\rangle_{a1}|V\rangle_{a2}|Hl\rangle_b\\&+|V\rangle_{a1}|H\rangle_{a2}|Vu\rangle_b+|V\rangle_{a1}|V\rangle_{a2}|Vl\rangle_b),\end{aligned} \tag{S6}$$

where photons $a1$ and $a2$ are two-dimensional and only have the polarization degree of freedom (DoF), and photon b is four-dimensional and has both the polarization and spatial DoF. If the polarization DoF and the spatial DoF of photon b are each regarded as a qubit separately, then this three-photon state can be rewritten as

$$\frac{1}{2}\left(|H\rangle_{a1}|H\rangle_{b.p}+|V\rangle_{a1}|V\rangle_{b.p}\right)\otimes\left(|H\rangle_{a2}|u\rangle_{b.s}+|V\rangle_{a2}|l\rangle_{b.s}\right), \tag{S7}$$

where b.p (b.s) corresponds to the polarization (spatial) DoF of photon b. From the above formula, it can be seen that this three-photon state can be viewed as a tensor product of two two-qubit maximally-entangled states. Since the two-qubit maximally entangled state $\frac{1}{\sqrt{2}}(|0\rangle|0\rangle+|1\rangle|1\rangle)$ can be expanded with the Pauli matrices as $\frac{1}{4}(XX-YY+ZZ+II)$, the prepared three-photon state can thus be expressed in terms of the Pauli matrix as

$$\left(X_{a1}X_{b.p}-Y_{a1}Y_{b.p}+Z_{a1}Z_{b.p}+I_{a1}I_{b.p}\right)\otimes\left(X_{a2}X_{b.s}-Y_{a2}Y_{b.s}+Z_{a2}Z_{b.s}+I_{a2}I_{b.s}\right). \tag{S8}$$

According to the above formula, we have measured the prepared three-photon state in the following nine bases $X_{a1}X_{a2}X_{b.p}X_{b.s}$, $X_{a1}Y_{a2}X_{b.p}Y_{b.s}$, $X_{a1}Z_{a2}X_{b.p}Z_{b.s}$, $Y_{a1}X_{a2}Y_{b.p}X_{b.s}$, $Y_{a1}Y_{a2}Y_{b.p}Y_{b.s}$, $Y_{a1}Z_{a2}Y_{b.p}Z_{b.s}$, $Z_{a1}X_{a2}Z_{b.p}X_{b.s}$, $Z_{a1}Y_{a2}Z_{b.p}Y_{b.s}$ and $Z_{a1}Z_{a2}Z_{b.p}Z_{b.s}$. Based on these measurement results, the fidelity of the prepared three-photon state with respect to the ideal (2, 2, 4) state can then be calculated.

S5. EXPERIMENTAL RESULTS OF QUANTUM STATE TRANSFER FROM TWO QUBITS TO A QUQUART

Figure S4 displays the experimental results for quantum state transfer of the six two-qubit states $|\varphi_1\rangle$-$|\varphi_6\rangle$.

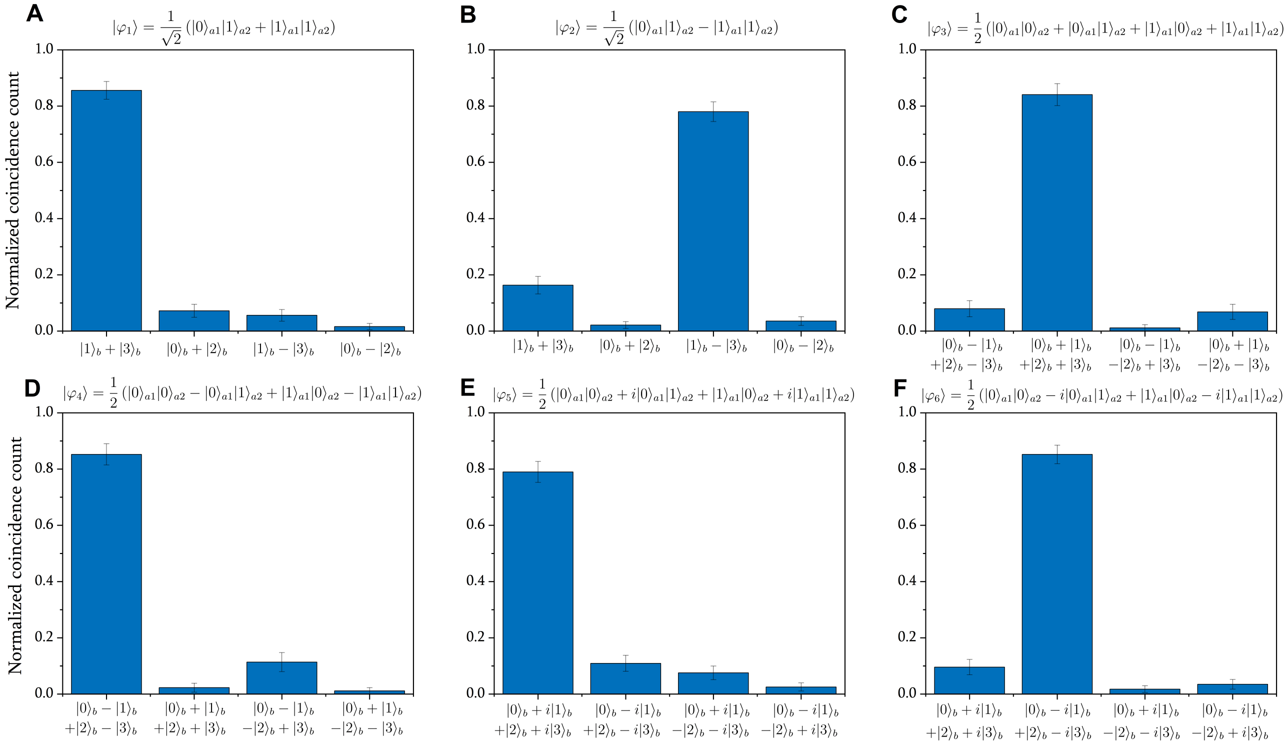

FIG. S4: Measurement results of the final ququart state for the two-qubit states $|\varphi_1\rangle$, $|\varphi_2\rangle$, $|\varphi_3\rangle$, $|\varphi_4\rangle$, $|\varphi_5\rangle$, $|\varphi_6\rangle$. For qubits $a1$ and $a2$, $|H\rangle$ and $|V\rangle$ are defined as $|0\rangle$ and $|1\rangle$, respectively. For photon b, $|Hu\rangle$, $|Hl\rangle$, $|Vu\rangle$ and $|Vl\rangle$ are defined as $|0\rangle$, $|1\rangle$, $|2\rangle$ and $|3\rangle$, respectively.

S6. SCHEME FOR PREPARING HIGH-DIMENSIONAL ASYMMETRIC ENTANGLED STATES USING INTEGRATED PHOTONIC CHIPS

In the main text, we introduced a method for realizing the (2,2,4) quantum state in discrete optical systems. Here, we extend this approach to integrated optical systems. As depicted in Fig. S5A, the input side of the integrated optical chip hosts four photons, individually labeled as $a1, a1', a2$, and b. Photon b is distributed across two waveguides marked in red. Photons $a1$ and $a1'$ form an entangled pair with the state $\frac{1}{\sqrt{2}}(|0\rangle_{a1}|0\rangle_{a1'} + |1\rangle_{a1}|1\rangle_{a1'})$, where 0 indicates the upper and 1 the lower waveguide. Similarly, $a2$ and b form another entangled pair with the state $\frac{1}{\sqrt{2}}(|0\rangle_{a2}|0\rangle_b + |1\rangle_{a2}|1\rangle_b)$. The optical chip contains two specific optical circuit modules (indicated by green frames), capable of implementing path-encode CNOT operations (*37*).

After photon $a1$ and photon b's upper path, and photon $a1'$ and photon b's lower path pass through the optical circuit modules, the four-photon quantum state becomes

$$\begin{aligned}&\frac{1}{2}(|0\rangle_{a1}|0\rangle_{a1'}|0\rangle_{a2}|0\rangle_b + |0\rangle_{a1}|0\rangle_{a1'}|1\rangle_{a2}|1\rangle_b \\ &+ |1\rangle_{a1}|1\rangle_{a1'}|0\rangle_{a2}|2\rangle_b + |1\rangle_{a1}|1\rangle_{a1'}|1\rangle_{a2}|3\rangle_b),\end{aligned} \tag{S9}$$

where the encoding scheme of photon b is shown in Fig. S5A. By projecting photon $a1'$ onto $\frac{1}{\sqrt{2}}(|0\rangle + |1\rangle)$, photons $a1, a2$ and b can be prepared into

$$\frac{1}{2}(|0\rangle_{a1}|0\rangle_{a2}|0\rangle_b + |0\rangle_{a1}|1\rangle_{a2}|1\rangle_b + |1\rangle_{a1}|0\rangle_{a2}|2\rangle_b + |1\rangle_{a1}|1\rangle_{a2}|3\rangle_b), \tag{S10}$$

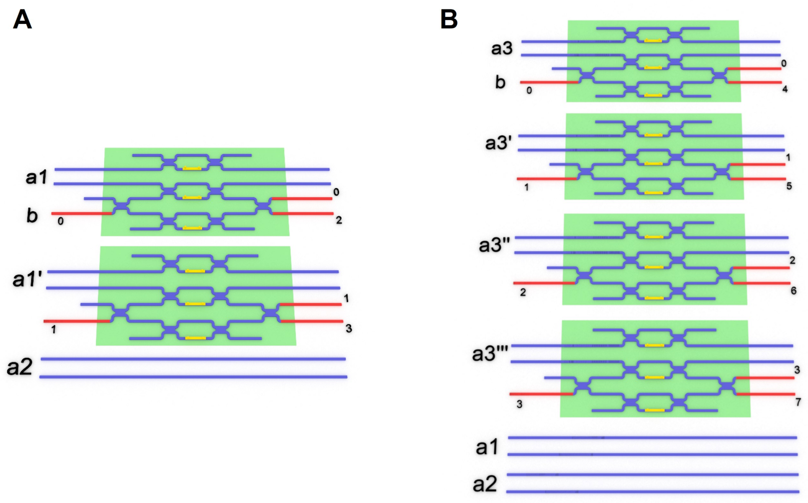

FIG. S5: Schematic of integrated optical chips for asymmetric entangled state preparation. (**A**) chip schematic for preparing the (2,2,4) state. (**B**) chip schematic for preparing the (2,2,2,8) state.

which is the desired (2, 2, 4) state.

The previously demonstrated method for preparing the (2,2,4) quantum state on an integrated optical chip can easily be expanded to higher dimensions. For instance, to illustrate the preparation of higher-dimensional asymmetric quantum states, we consider the (2,2,2,8) quantum state. As depicted in Fig. S5B, seven photons labeled $a3, a3', a3'', a3''', a1, a2$, and b are introduced at the input. Here, $a3$ through $a3'''$ comprise a four-photon entangled state $\frac{1}{\sqrt{2}}(|0\rangle_{a3}|0\rangle_{a3'}|0\rangle_{a3''}|0\rangle_{a3'''} + |1\rangle_{a3}|1\rangle_{a3'}|1\rangle_{a3''}|1\rangle_{a3'''})$, which can be prepared via the path identity method (*38*). Additionally, photons $a1, a2$, and b have already been configured into the (2,2,4) state using the previously described method. The optical chip includes optical circuit modules (indicated by green frames) identical to the earlier descriptions. After photon $a3$ and photon b's first path, photon $a3'$ and photon b's second path, photon $a3''$ and photon b's third path, and photon $a3'''$ and photon b's fourth path pass through the optical circuit modules, the quantum state of these seven photons becomes

$$\begin{aligned}\frac{1}{2\sqrt{2}}(&|0\rangle_{a3}|0\rangle_{a3'}|0\rangle_{a3''}|0\rangle_{a3'''}|0\rangle_{a1}|0\rangle_{a2}|0\rangle_{b} + |0\rangle_{a3}|0\rangle_{a3'}|0\rangle_{a3''}|0\rangle_{a3'''}|0\rangle_{a1}|1\rangle_{a2}|1\rangle_{b}\\ &+|0\rangle_{a3}|0\rangle_{a3'}|0\rangle_{a3''}|0\rangle_{a3'''}|1\rangle_{a1}|0\rangle_{a2}|2\rangle_{b} + |0\rangle_{a3}|0\rangle_{a3'}|0\rangle_{a3''}|0\rangle_{a3'''}|1\rangle_{a1}|1\rangle_{a2}|3\rangle_{b}\\ &+|1\rangle_{a3}|1\rangle_{a3'}|1\rangle_{a3''}|1\rangle_{a3'''}|0\rangle_{a1}|0\rangle_{a2}|4\rangle_{b} + |1\rangle_{a3}|1\rangle_{a3'}|1\rangle_{a3''}|1\rangle_{a3'''}|0\rangle_{a1}|1\rangle_{a2}|5\rangle_{b}\\ &+|1\rangle_{a3}|1\rangle_{a3'}|1\rangle_{a3''}|1\rangle_{a3'''}|1\rangle_{a1}|0\rangle_{a2}|6\rangle_{b} + |1\rangle_{a3}|1\rangle_{a3'}|1\rangle_{a3''}|1\rangle_{a3'''}|1\rangle_{a1}|1\rangle_{a2}|7\rangle_{b},\end{aligned} \tag{S11}$$

where the encoding scheme of photon b is shown in Fig. S5B. By projecting photon $a3', a3''$ and $a3'''$ onto $\frac{1}{\sqrt{2}}(|0\rangle+|1\rangle)$, photons $a1, a2, a3$ and b can be prepared into

$$\begin{aligned}\frac{1}{2\sqrt{2}}(&|0\rangle_{a3}|0\rangle_{a1}|0\rangle_{a2}|0\rangle_{b} + |0\rangle_{a3}|0\rangle_{a1}|1\rangle_{a2}|1\rangle_{b} + |0\rangle_{a3}|1\rangle_{a1}|0\rangle_{a2}|2\rangle_{b} + |0\rangle_{a3}|1\rangle_{a1}|1\rangle_{a2}|3\rangle_{b}\\ &+|1\rangle_{a3}|0\rangle_{a1}|0\rangle_{a2}|4\rangle_{b} + |1\rangle_{a3}|0\rangle_{a1}|1\rangle_{a2}|5\rangle_{b} + |1\rangle_{a3}|1\rangle_{a1}|0\rangle_{a2}|6\rangle_{b} + |1\rangle_{a3}|1\rangle_{a1}|1\rangle_{a2}|7\rangle_{b},\end{aligned} \tag{S12}$$

which is the desired (2, 2, 2, 8) state.

S7. QUANTUM GATE TRANSFER BASED ON ASYMMETRIC MAXIMALLY ENTANGLED STATE

In the emerging fields of quantum networks and distributed quantum computing, the inter-node capabilities need to extend beyond simple quantum state transfer to include quantum gate transfer. When nodes feature quantum information carriers of identical dimensions, gate teleportation—a technique previously validated in Ref. (*39*)—facilitates this remote operation. Conversely, if the dimensions of the quantum carriers at each node differ, the method for transferring quantum gates between these nodes remains undefined. This section details the process of transferring quantum gates between two-dimensional and four-dimensional systems through the use of asymmetric maximally entangled states. For instance, consider Alice's node equipped with two-dimensional quantum states (qubits) and Bob's with four-dimensional quantum states (ququarts). Bob aims to transfer a four-dimensional quantum gate, U, to Alice, enabling its application on Alice's pair of qubits. An asymmetric entangled (2,2,4) state serves as the medium to achieve this quantum gate transfer.

As shown in Fig. S6, the four-dimensional ququart b at Bob's node and two two-dimensional qubits $a1$ and $a2$ form an asymmetric entangled (2, 2, 4) state, where qubits $a1$ and $a2$ are sent to Alice's node. At Alice's node, two qubits $c1$ and $c2$ are in the state $|\psi\rangle_{c1,c2} = \alpha|00\rangle_{c1,c2} + \beta|01\rangle_{c1,c2} + \gamma|10\rangle_{c1,c2} + \delta|11\rangle_{c1,c2}$, which will serve as the operational target of the transferred quantum gate. Consequently, the quantum state comprising qubits $c1, c2, a1, a2$ and ququart b can be written as

$$\frac{1}{2}\left(|00\rangle_{a1,a2}|0\rangle_b + |01\rangle_{a1,a2}|1\rangle_b + |10\rangle_{a1,a2}|2\rangle_b + |11\rangle_{a1,a2}|3\rangle_b\right) \otimes \left(\alpha|00\rangle_{c1,c2} + \beta|01\rangle_{c1,c2} + \gamma|10\rangle_{c1,c2} + \delta|11\rangle_{c1,c2}\right). \tag{S13}$$

Two CNOT gates are then applied separately on qubit $c1$ and $a1$, as well as on qubit $c2$ and $a2$. Following these operations, qubits $a1$ and $a2$ are measured in the $|0\rangle/|1\rangle$ basis, and the measurement results are sent to Bob through a classical channel. When the measurement result of qubit $a1$ is 1, Bob performs an X_4 operation on ququart b. This operation swaps the states between components 0 and 2, as well as between 1 and 3. The matrix representation of this X_4 operation is

$$X_4 = \begin{pmatrix} 0 & 0 & 1 & 0 \\ 0 & 0 & 0 & 1 \\ 1 & 0 & 0 & 0 \\ 0 & 1 & 0 & 0 \end{pmatrix}. \tag{S14}$$

When the measurement result of qubit $a2$ is 1, Bob performs an X_4' operation on ququart b. This operation swaps the states between components 0 and 1, as well as between 2 and 3. The matrix representation of X_4' operation is

$$X_4' = \begin{pmatrix} 0 & 1 & 0 & 0 \\ 1 & 0 & 0 & 0 \\ 0 & 0 & 0 & 1 \\ 0 & 0 & 1 & 0 \end{pmatrix}. \tag{S15}$$

After these operations, the quantum state consisting of ququart b along with qubits $c1$ and $c2$ is transformed into

$$\alpha|0\rangle_b|00\rangle_{c1,c2} + \beta|1\rangle_b|01\rangle_{c1,c2} + \gamma|2\rangle_b|10\rangle_{c1,c2} + \delta|3\rangle_b|11\rangle_{c1,c2}. \tag{S16}$$

Ququart b is then subjected to a four-dimensional quantum gate U, designated for quantum gate transfer, followed by an operation with a H_4 gate, in which

$$U = \begin{pmatrix} e^{i\theta_0} & 0 & 0 & 0 \\ 0 & e^{i\theta_1} & 0 & 0 \\ 0 & 0 & e^{i\theta_2} & 0 \\ 0 & 0 & 0 & e^{i\theta_3} \end{pmatrix}, H_4 = \frac{1}{2}\begin{pmatrix} 1 & 1 & 1 & 1 \\ 1 & -1 & 1 & -1 \\ 1 & 1 & -1 & -1 \\ 1 & -1 & -1 & 1 \end{pmatrix} \tag{S17}$$

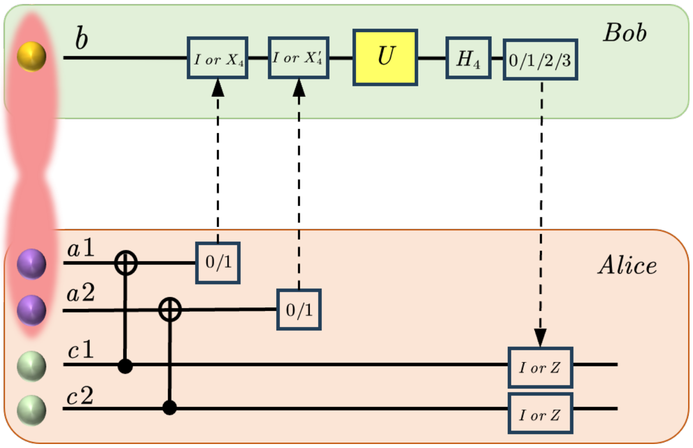

FIG. S6: Schematic for quantum gate transfer. Ququart b at Bob's node and two qubits a1 and a2 form an asymmetric entangled state (2, 2, 4), and qubits a1 and a2 are sent to Alice. At Alice's node, there are two qubits c1 and c2, whose initial state is $|\psi\rangle_{c1,c2}$. At Bob's node, there is a four-dimensional quantum gate U. After a series of operations as shown in the figure, the quantum state of qubits c1 and c2 are transformed into $U|\psi\rangle_{c1,c2}$, effectively accomplishing the transfer of quantum gate U from Bob to Alice.

Following these operations, the quantum state of qubit $c1, c2$, and ququart b becomes

$$\begin{aligned}
&\frac{1}{2}[(|0\rangle_b + |1\rangle_b + |2\rangle_b + |3\rangle_b)\alpha e^{i\theta_0}|00\rangle_{c1,c2} \\
&+(|0\rangle_b - |1\rangle_b + |2\rangle_b - |3\rangle_b)\beta e^{i\theta_1}|01\rangle_{c1,c2} \\
&+(|0\rangle_b + |1\rangle_b - |2\rangle_b - |3\rangle_b)\gamma e^{i\theta_2}|10\rangle_{c1,c2} \\
&+(|0\rangle_b - |1\rangle_b - |2\rangle_b + |3\rangle_b)\delta e^{i\theta_3}|11\rangle_{c1,c2}] \\
=&\frac{1}{2}[|0\rangle_b(\alpha e^{i\theta_0}|00\rangle_{c1,c2} + \beta e^{i\theta_1}|01\rangle_{c1,c2} + \gamma e^{i\theta_2}|10\rangle_{c1,c2} + \delta e^{i\theta_3}|11\rangle_{c1,c2}) \\
&+|1\rangle_b(\alpha e^{i\theta_0}|00\rangle_{c1,c2} - \beta e^{i\theta_1}|01\rangle_{c1,c2} + \gamma e^{i\theta_2}|10\rangle_{c1,c2} - \delta e^{i\theta_3}|11\rangle_{c1,c2}) \\
&+|2\rangle_b(\alpha e^{i\theta_0}|00\rangle_{c1,c2} + \beta e^{i\theta_1}|01\rangle_{c1,c2} - \gamma e^{i\theta_2}|10\rangle_{c1,c2} - \delta e^{i\theta_3}|11\rangle_{c1,c2}) \\
&+|3\rangle_b(\alpha e^{i\theta_0}|00\rangle_{c1,c2} - \beta e^{i\theta_1}|01\rangle_{c1,c2} - \gamma e^{i\theta_2}|10\rangle_{c1,c2} + \delta e^{i\theta_3}|11\rangle_{c1,c2})].
\end{aligned} \tag{S18}$$

Subsequently, measurements are conducted on ququart b on the basis of $|0\rangle/|1\rangle/|2\rangle/|3\rangle$. Depending on the outcome, specific operations are applied: a Z operation on qubit $c2$ for a result of 1, on qubit $c1$ for a result of 2, and on both qubit $c1$ and $c2$ for a result of 3. These operations transform qubits $c1$ and $c2$ into the state $\alpha e^{i\theta_0}|00\rangle_{c1,c2} + \beta e^{i\theta_1}|01\rangle_{c1,c2} + \gamma e^{i\theta_2}|10\rangle_{c1,c2} + \delta e^{i\theta_3}|11\rangle_{c1,c2} = U|\psi\rangle_{c1,c2}$, thus effectively accomplishing the transfer of quantum gate U from Bob to Alice, as the final state of qubits $c1$ and $c2$ corresponds to the action of U on the initial state $|\psi\rangle_{c1,c2}$.

It is important to note that the method described above applies specifically to scenarios where the quantum gate U is diagonal. For transferring more complex quantum gates, one could follow these steps: initially, qubits $c1$ and $c2$ at Alice's node are transferred onto a ququart at Bob's node by using an asymmetric (2, 2, 4) state. The quantum gate is then applied to this ququart. Following this, another asymmetric (2, 2, 4) state is used to transfer the modified ququart state back to the two qubits at Alice's node. This process effectively accomplishes the quantum gate transfer between Alice and Bob.
